# Root Extract of *Polygonum cuspidatum Siebold & Zucc.* Ameliorates DSS-Induced Ulcerative Colitis by Affecting NF-kappaB Signaling Pathway in a Mouse Model via Synergistic Effects of Polydatin, Resveratrol, and Emodin

**DOI:** 10.3389/fphar.2018.00347

**Published:** 2018-04-11

**Authors:** Baohai Liu, Shuangdi Li, Xiaodan Sui, Lianyi Guo, Xingmei Liu, Hongmei Li, Leming Gao, Shusheng Cai, Yanrong Li, Tingting Wang, Xuehua Piao

**Affiliations:** ^1^Department of Gastroenterology, The First Affiliated Hospital, Jinzhou Medical University, Jinzhou, China; ^2^Heart Disease Center, The Affiliated Hospital of Changchun University of Traditional Chinese Medicine, Changchun, China; ^3^Department of Hepatology, The Affiliated Hospital of Changchun University of Traditional Chinese Medicine, Changchun, China; ^4^The Second Dental Center, School of Stomatology, Peking University, Beijing, China; ^5^Department of Traditional Chinese Medicine, The First Affiliated Hospital of Jinzhou Medical University, Jinzhou, China

**Keywords:** ulcerative colitis, *Polygonum cuspidatum Siebold & Zucc.*, NF-κB signaling pathways, C57BL/6J mouse, inflammatory cytokine

## Abstract

**Background:** *Polygonum cuspidatum Siebold & Zucc.* (PCS) has antibacterial properties and may prevent Ulcerative colitis (UC) but related molecular mechanism remains unknown. NF-κB signaling pathway is associated with inflammatory responses and its inactivation may be critical for effective therapy of UC.

**Methods:** UC mouse (C57BL/6J) model was established by using dextran sulfate sodium (DSS). The extract of PCS (PCSE) was prepared by using ethanol and its main ingredients were measured by HPLC. Thirty-two UC mice were evenly assigned into DG (received vehicle control), LG (0.1 g/kg PCSE daily), MG (0.2 g/kg PCSE daily) and HG (0.4 g/kg PCSE daily) groups. Meanwhile, 8 healthy mice were assigned as a control group (CG). Serum pharmacokinetics of PCS was measured by using HPLC. After 8-day treatment, weight, colon length and disease activity index (DAI) were measured. Inflammatory cytokines and oxidant biomarkers were measured by ELISA kits. The levels of cytokines, and key molecules in NF-κB pathway, were measured by using Western Blot. The effects of main ingredients of PCSE on cytokines and NF-κB signaling pathway were explored by using intestinal cells of a mouse UC model. The normality criterion was evaluated using the Saphiro–Wilk test. The quantitative variables were compared using the paired Student’s-*t* test.

**Results:** The main ingredients of PCSE were polydatin, resveratrol and emodin. Polydatin may be transformed into resveratrol in the intestine of the mice. PCSE prevented DSS-caused weight loss and colon length reduction, and improved histopathology of UC mice (*P* < 0.05). PCSE treatment increased the serum levels of IL-10 and reduced the levels of IL-1 beta, IL-6 and TNF-α (*P* < 0.05). PCSE increased the activities of SOD, CAT, GPX and reduced the level of MDA, BCL-2, beta-arrestin, NF-κB p65 and the activity of MPO (*P* < 0.05). The combination of polydatin, resveratrol or emodin, and or PCSE exhibited higher inhibitory activities for cytokines and NF-κB signaling related molecules than any one of the three ingredients with same concentration treatment.

**Conclusion:** Oral administration of PCSE suppressed NF-κB signaling pathway and exerts its anti-colitis effects via synergistic effects of polydatin, resveratrol or emodin.

## Introduction

Ulcerative colitis (UC) is a common repeated and prolonged inflammatory disease of digestive system. Its pathological manifestations are diverse, including abdominal pain (Castelli et al., 2018), diarrhea (Zhong et al., 2017), and tenesmus (Nigg et al., 2008) etc. The exact pathogenesis of UC has not been clarified yet. The abnormalities in immunity and genetic susceptibility are the main research objects in recent years. Several drugs may be effective in treating UC but all the drugs have remarkable side effects, which significantly inhibit their clinical use. However, maintaining a good mucosal state is still important for maintaining remission (Suzuki et al., 2017). Although mesalamine has been used as the first-line medicine for UC therapy as an effective drug, earlier research demonstrated that mesalamine could cause diarrhea (Shimodate et al., 2011). Another example, corticosteroid beclomethasone dipropionate (BDP) and prednisone (PD) were safe and effective in treating the patients with active and mild-to-moderate UC (Van Assche et al., 2015a). However, patients had a high prevalence of oral corticosteroid (OCS) complications including trouble sleeping and weight gain (Van Assche et al., 2015b). PD induces neuropsychological side effects, which reduce life quality of patients (Warris et al., 2014). Furthermore, drug resistance due to the extensive abuse of antibiotics is a serious issue, and exploring alternative antibiotics has become very urgent. Therefore, it is necessary to explore new effective medicine with fewer side effects.

*Polygonum cuspidatum Siebold & Zucc.* is a natural plant that is widely distributed in China and Japan, and it is used in traditional Chinese herbal medicine. *P. cuspidatum Siebold & Zucc.* is rich with phenolics and has been used for the treatments of amenorrhea, arthralgia, jaundice, abscess, scald, and bruises (Lin et al., 2012). The main active ingredients in *P. cuspidatum Siebold & Zucc.* are epicatchin, resveratrol, and emodin according to HPLC-diode array detection-flow injection-chemiluminescence (Ding et al., 2013). Epicatchin treatment has been reported to ameliorate the toxicity of cyclosporine A by decreasing the lipid peroxidation and enhancing the antioxidant properties by reducing the levels of reactive oxygen species (Al-Malki and Moselhy, 2011). Previous data demonstrated that resveratrol exerted its protective functions by mediating oxidative stress and enhancing antioxidant activities. It also can the pathological changes in animals against inflammation caused by nicotine (Hamza and El-Shenawy, 2017). Emodin can protect cells against the damage caused by cerulein and lipopolysaccharide and control inflammatory status in cells (Zhao et al., 2017). The extracts from *P. cuspidatum Siebold & Zucc.* (PCSE) have a promising antimicrobial activity for controlling drug-resistant bacteria (Su et al., 2015). However, the effects of PCSE on UC and related molecular mechanism remain unknown.

Nuclear factor-κB (NF-κB) p65 is an important transcription factor, which regulates a number of genes associated with immune and inflammatory responses. NF-κB signaling pathway responds to various stimuli, such as TNF receptor (TNFR), T-cell receptor (TCR), and B-cell receptor. NF-κB pathway also responds to specific stimuli, including LTβR, BAFFR, CD40 and RANK. NF-κB regulates the activation and differentiation of T cells and inflammasomes (Chi et al., 2015; Gan et al., 2016). NF-κB p65 is a pivotal transcription factor of M1 macrophages and induces a great amount of inflammatory genes, including TNF-α, IL-1β, IL-6, IL-10, IL-12 and cyclooxygenase-2 (Jeoung et al., 2013; Jeong et al., 2017). Inactivation of the NF-κB, Stat3 is related to the potential pro-apoptotic signaling pathways (Xiang et al., 2017). NF-κB will increase BCL-2 expression, which leads to a decrease in cellular apoptosis (Xie et al., 2017). Beta-arrestin is associated with the activation of NF-κB pathway, localizing transcription factors in nuclei and initiating COX-2 expression, thereby linking internalization of the receptors with the NF-κB pathway (Morinelli et al., 2013). Previous studies showed NF-κB signaling dysfunction in the pathogenesis and progression of UC (Eissa et al., 2017; Gu et al., 2017). NF-κB signaling pathway has been widely reported to be contributed to inflammatory responses (Pan et al., 2017; Ding et al., 2017). Therefore, inactivation of NF-κB signaling pathway may be required is of great significance for effective therapy of UC. Resveratrol, the most important ingredient extracted from *P. cuspidatum*, exhibits anti-inflammatory function by affecting NF-κB signaling pathways(Ma et al., 2015). This study aimed to explore the effects of PCSE on UC by investigating NF-κB signaling pathway.

## Materials and Methods

### PCSE Preparation

*Polygonum cuspidatum Siebold & Zucc.* was purchased from the Guilin Pharmaceuticals (Guilin, China). PCES was obtained by using the roots and rhizomes of *P. cuspidatum Siebold & Zucc.* based on “Pharmacopoeia of the People’s Republic of China” (2010 version). Polyphenolic compounds resveratrol and glycosides are the main composition of the *P. cuspidatum Siebold & Zucc.*(Yang et al., 2001). However, the Pharmacopeia his does not specify the contents of resveratrol in PCSE. The roots of *P. cuspidatum Siebold & Zucc.* were ground and 100 g of ground power was extracted with one liter of 95% ethanol in a Soxhlet apparatus (Udhna, Surat, Gujarat, India). The liquid was evaporated on rotavapour (Buchi, New Castle, DE, United States) at 65°C. PCSE was finally dried by using a vacuum freeze drier (SCIENTZ-18N, Shanghai, China). The yields of PCSE were 16.9 ± 1.7% of dried PCS.

### HPLC Analysis of PCSE

The standards of resveratrol, polydatin and emodin were purchased from Sigma Chemical (St. Louis, MO, United States). The main components of PCSE were measured by using high performance liquid chromatography (HPLC, Waters Associates, Milford, MA, United States) with a Waters Acquity BEH300 C18 column (2.1 mm × 100 mm, 1.7 μm), a PDA detector, and Empower2 chromatography manager software. The column temperature was 35°C, the flow rate was 0.3 mL/ min, and the detection wavelength was 280 nm. Mobile phase A was 0.5% acetic acid and B was acetonitrile. 10-μL sample was injected and gradient elution was as follows: 0–7 min, 8–20% B; 7–12 min, 20–40% B; 12–16 min, 40–60% B; 16–20 min, 60–65% B; 20–24 min, 65–95% B; 24-30 min, 95% B.

### Animals

All processes were approved by the Animal Research Ethical Committee of Jinzhou Medical University (Approval No. 20160308x, Jinzhou, China). A total of 40 male mice (C57BL/6J, 6 weeks old) weighing 18–22 g were purchased from Experimental Animal Center of Jinzhou Medical University (License No. SCXK2014-0002; animal qualified certificate No. SCXK2014-0004). Mice were kept under an automated 12-h light-dark cycle at a controlled temperature of 22 ± 2°C, relative humidity of 50-60% and had *ad libitum* access to a standard dry diet and tap water. The animals received humane care and experimental procedures were performed in accordance with the health and care of experimental animals’ guidelines of the Jinzhou Medical University (Jinzhou, China).

### Induction of Colitis

DSS induced colitis was performed as previously described method (Castelli et al., 2018). DSS was purchased from Sigma–Aldrich and added to drinking water at a final concentration of 3%. The mice received the drinking water for a week. Subsequently, the mice received tap water for 5 days. The DSS solution was made freshly and changed daily. Mice were randomly divided into five groups (control group, colitis control group and three experimental groups) with eight in each group. Mice were given drinking water in control group and drinking water containing 4% DSS (MW 36000-50000, MP Biomedical) in colitis control group and three experimental groups for 7 days and then all of the mice were shifted to normal tap water in day 8. The animals were given free access to water during the experiment. The mice in normal control and colitis control groups were orally administrated with 0.5 mL phosphate-buffered saline. Three experimental groups were orally administrated with different dosage of PCSE (100, 200 mg/kg and 400 mg/kg) in 0.5 mL 0.85% NaCl saline for 8 days from the first day of induction DSS. The control and some model animals received vehicle with 0.85 NaCl salt solution alone.

### Animal Grouping

All animals were assigned into five groups according to different treatments: control (CG), model (DG), low-dose (LG), middle-dose (MG) and high-dose groups (HG) as **Table 1** showed. All groups had the diets with same energy density (3.78 kcal/g), macronutrient composition, and trace elements. All animas were monitored daily for the symptoms of colonic inflammation, bloody stool and weight loss. Disease activity index (DAI) was measured in accordance with previously reported (Murthy et al., 1993).

**Table 1:** Composition of experimental diets Ingredient (g/kg).

**Ingredients**	**CG**	**Ulcerative colitis models**			
		**DG**	**LG**	**MG**	**HG**
Atlantic salmon	200	200	200	200	200
Casein	35	35	35	35	35
L-cystine	3 3	3 3	3 3	3 3	3 3
Corn starch	386.5	386.5	386.5	386.5	386.5
Maltodextrin	132	132	132	132	132
Sucrose	100	100	100	100	100
Fruit and vegetable powder	60	60	60	60	60
Soybean oil	6	6	6	6	6
Cellulose	30	30	30	30	30
Mineral mix, AIN-93G-MX	35	35	35	35	35
Vitamin mix, AIN-93-VX	10	10	10	10	10
Choline bitartrate 2.5	2.5	2.5	2.5	2.5	2.5
tert-Butylhydroquinone	0.014	0.014	0.014	0.014	0.014
Polygonum Cuspidatum	–	–	0.1	0.2	0.4
Water	238	238	238	238	238

*All animals were assigned into five groups according to different treatments: control (CG), model (DG), low-dose (LG), middle-dose (MG) and high-dose groups (HG); *n* = 8 in each group.*

### Serum Pharmacokinetics Measurements

One-half milliliter of blood was obtained from mouse tail vein after 4-h PCSE treatment and serum was isolated by centrifuged at 2000 rpm for 5 min. Fifty-microliter serum was taken and 200-μL methanol was added. The mixture was centrifuged at 12,000 *g* × 10 min, passed through a 0.45 μm Millipore filter, and 10-μL sample was used for HPLC analysis.

### Biopsy Specimen Preparation

All mice were sacrificed by cervical dislocation on day 9. Colon biopsy specimens were obtained and opened longitudinally, and the fecal were removed. The specimens were washed with a 0.85% NaCl saline solution and immediately examined.

### Histology Analysis of Colonic Damage

After euthanasia, the colon was isolated and carefully cleaned of mesentery tissue, vessels and fat, and the length measured. A 4 cm segment of the distal colon was removed and weighed after rinsing the lumen with PBS. For the histological examination, 1 cm segments from the distal colon were fixed in 4% formalin, embeded in paraffin blocks and cut into 5 μm-thick sections and placed on glass slides. The sections were stained with hematoxylin and eosin (H & E) to study histological changes or with1% Alcian blue 8GX (Sigma) in 0.1 N HCl for 2 h to investigate. Sections were assessed in a blinded fashion. Stained colon sections were observed under Olympus microscope (Tokyo, Japan).

### Immunofluorescence

Above sections were fixed with acetone on ice for half an hour. After being washed three times by using PBS (50 mM, pH7.0), the samples were blocked with mouse IgG for 2 h at room temperature. Subsequently, they were incubated with FITC and (Thermo Fisher Scientific) and PE-CY5 anti-β-arrestin or anti-Bcl-2. Fluorescent density was observed at 40 magnifications under on an inverted fluorescent microscope (Nikon, Tokyo, Japan).

### Level of Inflammatory Factors in Mouse Serum

One-half milliliter of blood was obtained from mouse tail vein and serum was isolated by centrifuged at 2000 rpm for 5 min. The concentrations of IL-1β (Cat. No. ab197742), IL-6 (Cat. No. ab100713), IL-10 (Cat. No. ab46103) and TNF-α (Cat. No. ab46105) were measured by using ELISA kits from Abcam (Cambridge, MA, United States).

### Measurement of Oxidative Stress Markers

One-gram colon was mashed with mortar and pestle in 1 ml of 20 mM potassium phosphate buffer (pH 7.0). Supernatants were collected by centrifugation at 12,000 rpm for 10 min. The activity of Superoxide Dismutase (SOD) was measured by using SOD Activity Colorimetric Assay Kit from Dojindo Molecular Technologies, Inc. (Minato-ku, Tokyo, Japan). The activities of Glutathione Peroxidase (GPx) (Cat. No. ab102530), Myeloperoxidase (MPO) (Cat. No. ab43321), Catalase (CAT) (Cat. No. ab83464), and Lipid Peroxidation (MDA) (Cat. No. ab118970) levels were measured by using Assay Kits from Abcam (Cambridge, MA, United States).

### Western Blot Analysis

Proteins were extracted from animal colon biopsy specimens by using protein isolation kit (Miltenyi Biotec Inc, Auburn, CA, United States). The samples were resolved on SDS-PAGE (12 gel), and blotted onto PVDF membranes (Millipore Corp., Bedford, MA, United States) blocked in 5% milk for 20 min. The membrane was incubated with rabbit-derived first antibodies of NF-κB p65 (Cat. No. ab16502), TNF-à (Cat. No. ab6671), β-arrestin (Cat. No. ab31868), BcL-2 (Cat. No. ab196495), IL-1β (Cat. No. ab82558), IL-6 (Cat. No. ab6672), IL-10 (Cat. No. ab9969) and GAPDH (Cat. No. ab37168), which were purchased from Abcam (Cambridge, MA, United States). The blot was labeled with Goat Anti-Rabbit IgG H&L (HRP) (Cat. No. ab6721 from Abcam). Signals were quantified by ImageJ (developed at the National Institutes of Health).

### The Effects of Resveratrol, Polydatin and Emodin on Mouse Intestinal Cells

One-gram small intestine of a UC model was also excised immediately and ground in a mortar and pestle with 1.5-mL 0.85% saline solution. The tissue fragments were placed in 2 mL sterile tubes, and digested by 0.25% trypsin and 0.1% collagenase type at 37°C for 30 min. Digested tissue was filtrated through a 100-μm screen and centrifuged at 1000 *g* for 5 min to get separated intestinal cells. The cells were adjusted to a density of 1 × 10^4^ cells/mL and inoculated with 100 μL high-glucose DMEM medium containing 10% fetal bovine serum, penicillin 100 IU/L, streptomycin 100 mg/L per well at 37°C in a 5% CO^2^ incubator. Standard resveratrol, polydatin and emodin were added to final 10 μg/mL with the medium. Two mixed groups (mixed group 1: 3.3 μg/mL of resveratrol, polydatin and emodin, respectively; PCSE, 13.3 μg/mL, total of resveratrol, polydatin and emodin was 10 μg/mL) were designed as positive controls. After 4 h, the culture medium was discarded. The cells were lysed by 100 μL of CelLytic Buffer (Sigma). Cytokine and NF-κB related molecules were measured by using ELISA kits. The ELISA kits for NF-κB p65 (Total) ELISA Kit (ab176648), IL-1β (Cat. No. ab197742), IL-6 (Cat. No. ab100713), IL-10 (Cat. No. ab46103), TNF-α (Cat. No. ab46105) and Bcl-2 (ab227899) were from Abcam (Cambridge, MA, United States). A mouse β-arrestin ELISA kit (MBS9356683) was from MyBioSource (San Diego, CA, United States).

### Statistical Analysis

All data were presented as mean ± standard derivative (S.D.). Statistical analysis was performed by using SPSS 20.0 (IBM, Armonk, NY, United States). The normality criterion was evaluated using the Saphiro–Wilk test. The quantitative variables were compared using the paired Student’s-*t* test. The statistical differences were significant if *P*-values < 0.05.

## Results

### The Main Ingredients of PCSE

**FIGURE 1:**
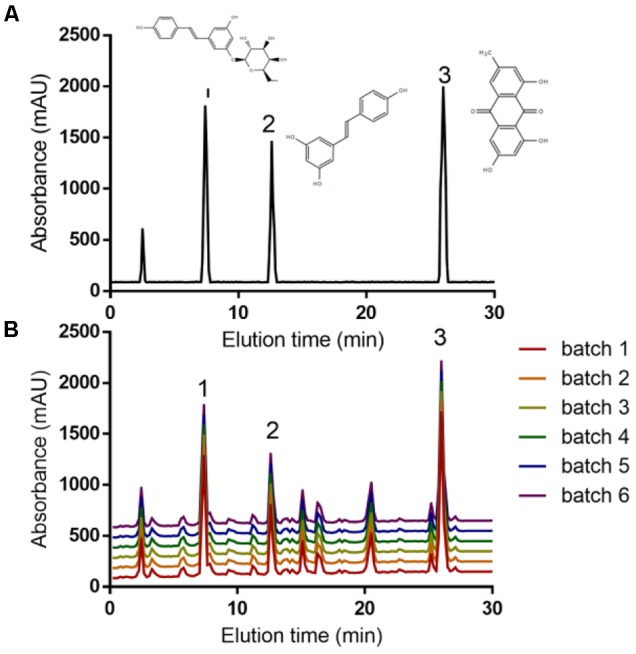
HPLC analysis of PCSE. **(A)** The standards of polydatin (peak 1), resveratrol (peak 2), emodin (peak 3). **(B)** The main components of PCSE from 6 batches.

HPLC analysis showed that the main components of PCSE were polydatin (peak 1), resveratrol (peak 2) and emodin (peak 3) (**Figure 1B**) according to the standards (**Figure 1A**). The contents of three components were stable from 6 batches (**Figure 1B**).

### Serum Pharmacokinetics of PCSE

**FIGURE 2:**
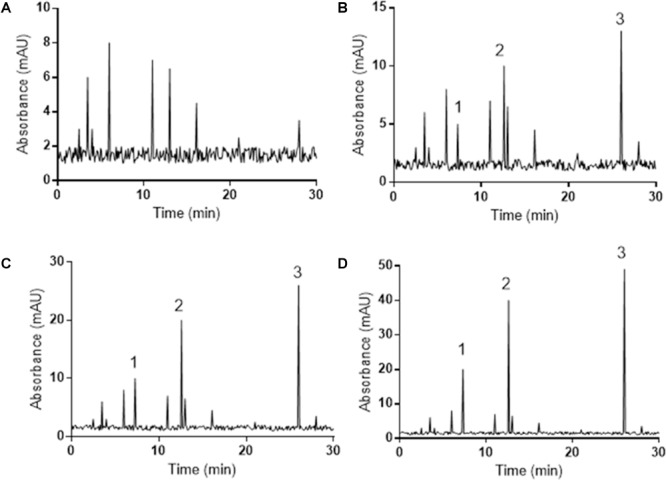
HPLC determination of serum pharmacokinetics measurements of PCSE in mouse UC models. All animals were assigned into five groups according to different dose of PCSE: model (DG, without PCSE, **A**), low-dose (LG., 0.1 g/kg PCSE, **B**), middle-dose (MG, 0.2 g/kg PCSE, **C**) and high-dose groups (HG, 0.4 g/kg PCSE, **D**).

Serum pharmacokinetics analysis showed that there were no polydatin, resveratrol or emodin in the serum of the mice from DG group (**Figure 2A**). Polydatin, resveratrol and emodin could be detected by HPLC in the serum of the mice from LG (**Figure 2B**), MG (**Figure 2C**) and HG (**Figure 2D**) groups. Meanwhile, the increase of the serum levels of three components was the same as the fold increase in PCSE addition (**Figure 2**).

### Effects of PCSE on Weight Loss in UC Mice

**FIGURE 3:**
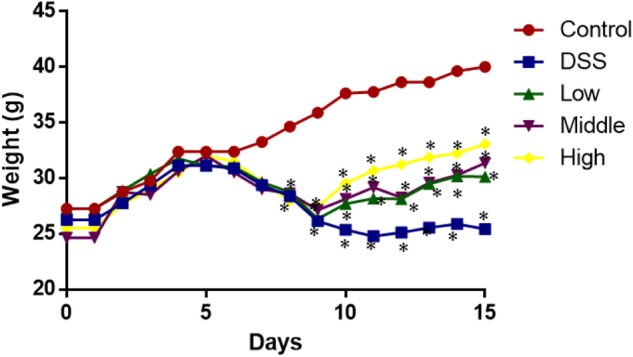
The effects of PCSE on the weight of mouse UC models. ^∗^*P* < 0.05 via a control group.

From day 0 to 5, the statistical differences in weight loss were insignificant among all the groups (**Figure 3**, *P* > 0.05). After 6 days, the weight loss became obvious between control group and other groups (**Figure 3**, *P* > 0.05). PCSE prevented weight loss in a mouse UC models when compared with the models without PCSE treatment. After 10 days, the statistical differences were significant between DG and HG groups (**Figure 3**, *P* > 0.05).

### Effects of PCSE on DSS-Caused Colon Length Reduction in UC Mice

**FIGURE 4:**
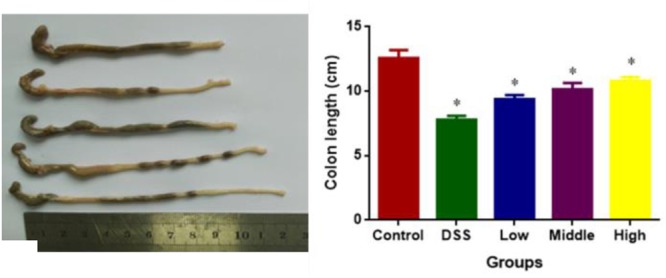
The effects of PCSE on DSS-caused colon length reduction in a mouse UC model. ^∗^*P* < 0.05 via a control group.

DSS treatment caused colon length reduction in a mouse UC models. The statistical differences were significant for colon length reduction between CG and other groups (**Figure 4**, *P* < 0.05). PCSE prevented DSS-caused colon length reduction in a mouse UC models, and the statistical differences were significant between DG and HG groups (**Figure 4**, *P* < 0.05).

### Effects of PCSE on Disease Activity Index (DAI) in UC Mice

**FIGURE 5:**
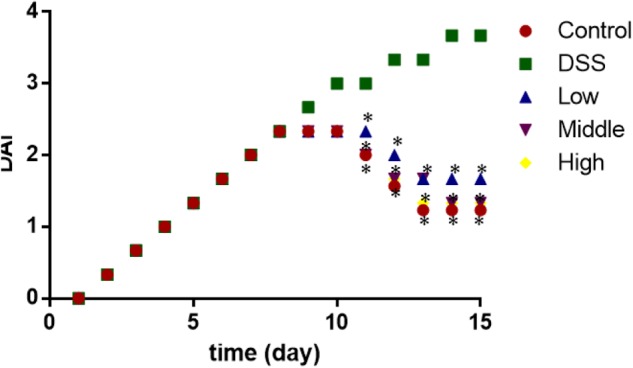
The effects of PCSE on disease activity index (DAI) in a mouse UC model. ^∗^*P* < 0.05 via a control group.

From day 0 to 8, the statistical differences for DAI were insignificant among all the groups (**Figure 5**, *P* > 0.05). After 8 days, the DAI became obvious between control group and other groups (**Figure 5**, *P* > 0.05). PCSE controlled the increase of DAI in a mouse UC models when compared with the mouse models without PCSE treatment. After 10 days, the statistical differences were significant between DG and HG groups (**Figure 5**, *P* < 0.05).

### Effects of PCSE on Protection Against the Colon Damage Caused by DSS

**FIGURE 6:**
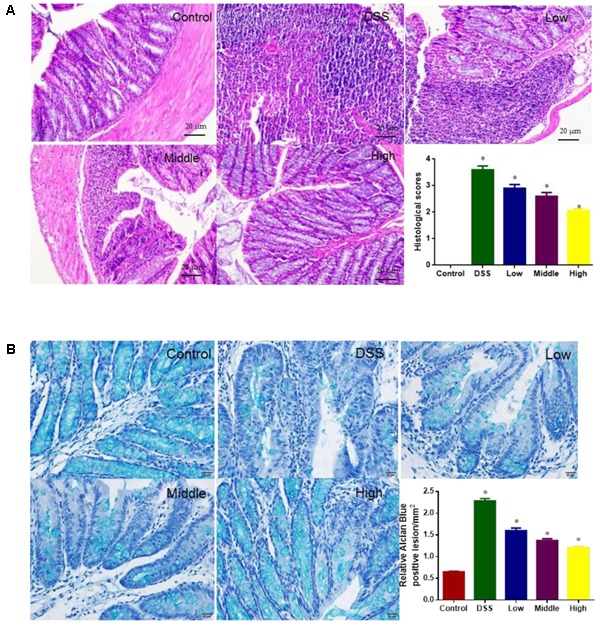
Histological analysis the effects of PCSE on the colon damage caused by DSS in a mouse UC model. **(A)** H & E staining analysis. **(B)** Alcian blue staining analysis. ^∗^*P* < 0.05 via a control group.

H & E analysis showed that tissue structure was in normal form with perfect cell nucleus and cell plasm (**Figure 6A**). DSS treatment caused tissue damage with destroyed cells whereas PCSE reduced the damage severity with the increase in its dosage. For histological scores, the statistical differences were significant between DG and HG groups (**Figure 6A**, *P* < 0.05).

Alcian blue stain showed that the Alcian blue staining of the colon was significantly diminished in DG and LG groups when compared with CG and HG (**Figure 6B**). Relative lesion areas were bigger in the DG group than CG group (*P* < 0.0). PCSE reduced relative lesion areas with the increase in its dosage.

### Effects of PCSE on the Serum Levels of Inflammatory Factors

**FIGURE 7:**
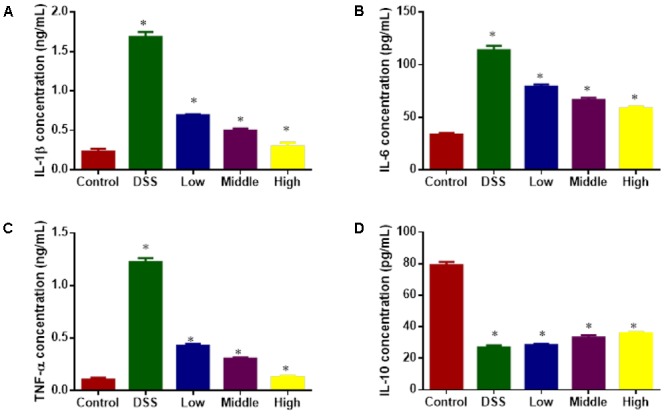
ELISA analysis of the effects of PCSE on serum levels of inflammatory factors in a mouse UC model. **(A)** The effects of PCSE on serum levels of IL-1β. **(B)** The effects of PCSE on serum levels of IL-6. **(C)** The effects of PCSE on serum levels of TNF-α. **(D)** The effects of PCSE on serum levels of IL-10. ^∗^*P* < 0.05 via a control group.

ELISA analysis showed that DSS treatment increased the serum levels of inflammatory factors IL-1β (**Figure 7A**), IL-6 (**Figure 7B**) and TNF-α (**Figure 7C**) and reduced the level of IL-10 (**Figure 7D**) when compared with healthy animals (*P* < 0.05). PCSE inhibited the serum levels of inflammatory factors IL-1β, IL-6 and TNF-α and increased the level of IL-10 with the increase in its dosage, and the statistical differences were significant between DG and HG groups (**Figure 7**, *P* < 0.05).

### Effects of PCSE on the Antioxidant Ability of UC Models

**FIGURE 8:**
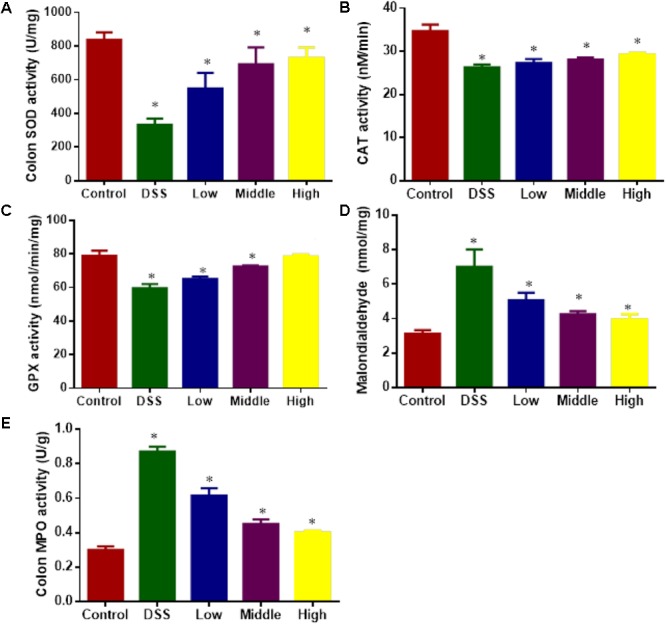
The effects of PCSE on oxidative biomarkers in a mouse UC model. **(A)** The effects of PCSE on the activity of SOD in a mouse UC model. **(B)** The effects of PCSE on the activity of CAT in a mouse UC model. **(C)** The effects of PCSE on the activity of GPX in a mouse UC model. **(D)** The effects of PCSE on the level of MDA in a mouse UC model. **(E)** the effects of PCSE on the activity of MPO in a mouse UC model. ^∗^*P* < 0.05 via a control group.

DSS treatment increased oxidative stress of UC models by reducing the levels SOD (**Figure 8A**), CAT (**Figure 8B**), and GPX (**Figure 8C**), increasing the levels of MDA (**Figure 8D**) and MPO (**Figure 8E**) when compared with healthy animals (*P* < 0.05). Comparatively, PCSE improved antioxidant capacities of UC mice by increasing the levels SOD (**Figure 8A**), CAT (**Figure 8B**), and GPX (**Figure 8C**), and reducing the levels of MDA (**Figure 8D**) and MPO (**Figure 8E**) with the increase of its dosage.

### Effects of PCSE on Regulating Key Molecules Involved in NF-κB Signaling Pathway

**FIGURE 9:**
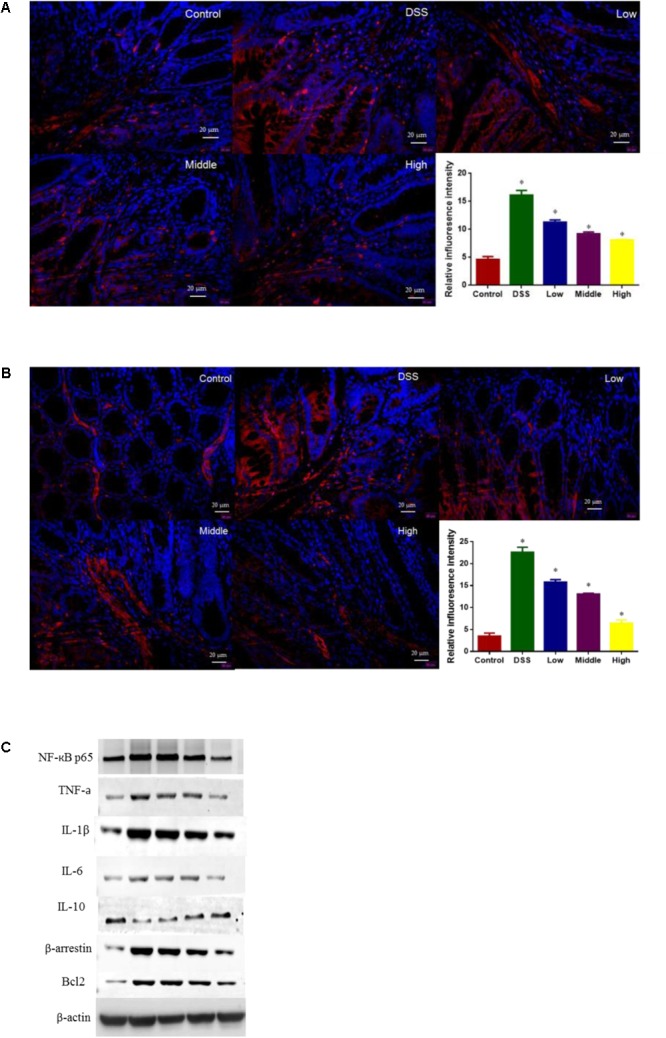
The effects of PCSE on the levels of key molecules involved in NF-κB signaling pathway. **(A)** Inflorescence intensity analysis of the level of BCL-2. **(B)** Inflorescence intensity analysis of the level of β-arrestin. **(C)** Western Blot analysis of the effects of PCSE on the levels of key molecules involved in NF-κB signaling pathway. ^∗^*P* < 0.05 via a control group.

In fluorescence analysis showed that DSS treatment increased the levels of BCL-2 (**Figure 9A**) and β-arrestin (**Figure 9B**) when compared with control group (*P* < 0.05). Western Blot analysis showed that DSS treatment reduced the level of IL-10 and increased the levels of NF-Kb p65, β-arrestin, BCL-2, IL-1 and IL-6 when compared with control group (**Figure 9C**, P < 0.05). Comparatively, PCSE inhibited inflammatory levels of UC mice by reducing the level of IL-10 and increasing the levels of IL-1 and IL-6 when compared with control group (**Figure 9C**, P < 0.05). Meanwhile, PCSE consumption prevented the activities of NF-Kb signaling pathway by reducing the levels of BCL-2 (**Figures 9A,C**), β-arrestin (**Figures 9B,C**) and NF-Kb.

### Effects of PCSE on Preventing Colon Apoptosis in a Mouse UC Model

**FIGURE 10:**
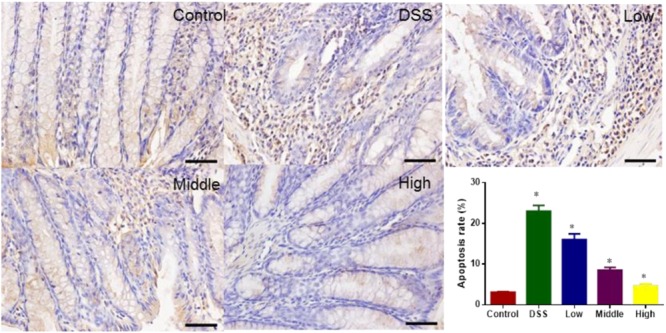
TUNEL analysis of the effects of PCSE on colon apoptosis of mouse UC model. ^∗^*P* < 0.05 via a control group. ^∗^*P* < 0.05 via a control group.

TUNEL analysis showed that DSS treatment increased colon apoptosis in a mouse UC models. The statistical differences were significant for apoptosis rates between CG and other groups (**Figure 10**, *P* < 0.05). PCSE prevented DSS-caused apoptosis in a mouse UC models, and the statistical differences were significant between DG and HG groups (**Figure 10**, *P* < 0.05).

### The Synergistic Effects of Polydatin, Resveratrol or Emodin on NF-kappaB Signaling

**FIGURE 11:**
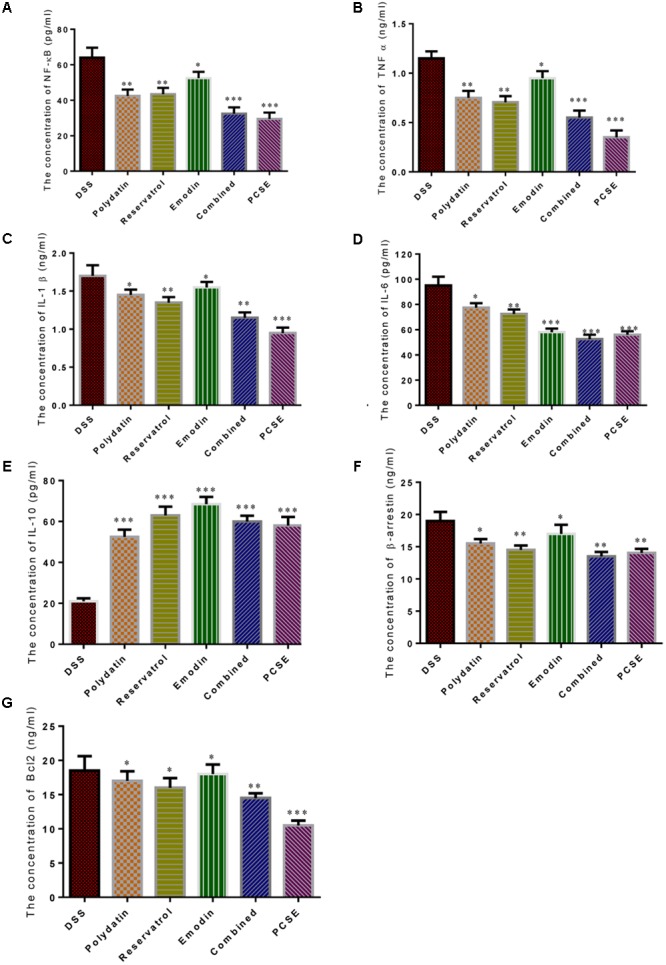
The effects of different components of PCSE on the levels of key molecules involved in NF-κB signaling pathway. **(A)** The concentration of NF-κB. **(B)** The concentration of TNF-α. **(C)** The concentration of IL-1β. **(D)** the concentration of IL-6. **(E)** The concentration of IL-10. **(F)** The concentration of βarrestin. **(G)** The concentration of Bcl2. Standard resveratrol, polydatin and emodin were added to final 10 μg/mL with medium. Two mixed groups (mixed group 1: 3.3 μg/mL of resveratrol, polydatin and emodin, respectively; mixed group 2: PCSE 13.3 μg/mL, total of resveratrol, polydatin and emodin was 10 μg/mL) were designed as positive controls. ^∗^*P* < 0.05 vs. a control group, ^∗∗^*P* < 0.01 vs. a DSS group and ^∗∗∗^*P* < 0.001 vs. a control group.

**Figure 11** showed that all the three main components polydatin, resveratrol or emodin could reduce the concentrations of NF-κb (**Figure 11A**), TNF-α (**Figure 11B**), IL-1β (**Figure 11C**), IL-6 (**Figure 11D**), β-arrestin (**Figure 11F**), and Bcl2 (**Figure 11G**), and increased the concentration of IL-10 (**Figure 11E**). The concentrations of NF-κb, TNF-α, IL-1β, IL-10,β-arrestin and Bcl2 were reduced (*P* < 0.05), and the concentration of IL-6 was significantly reduced (*P* < 0.01) by emodin when compared with DSS group. The concentrations of IL-1β, IL-6,β-arrestin and Bcl2 were reduced (*P* < 0.05), and the concentration of NF-κb and TNF-α was significantly reduced (*P* < 0.01) by polydatin when compared with DSS group. The concentrations of NF-κb, TNF-α, IL-1β, IL-6, andβ-arrestin were significantly reduced (*P* < 0.01), and the concentration of Bcl2 was reduced (*P* < 0.05) by resveratrol when compared with DSS group. The concentrations of NF-κb, TNF-α and IL-6 were very significantly reduced (*P* < 0.001), and the concentrations of IL-1β-arrestin and Bcl2 were significantly reduced (*P* < 0.01) by the combination of three components when compared with DSS group. The concentrations of NF-κb, TNF-α, IL-1β, IL-6, and Bcl2 were very significantly reduced (*P* < 0.01), and the concentration of β-arrestin was significantly reduced (*P* < 0.01) by PCSE when compared with DSS group. The inhibitory activities of the combination of polydatin, resveratrol or emodin, and PCSE were higher than any one of the three components with same concentration treatment. The results suggest that the synergistic effects of polydatin, resveratrol or emodin on NF-kappaB signaling.

## Discussion

The main ingredients of PCSE were polydatin, resveratrol and emodin. HPLC analysis showed that the serum level of resveratrol was higher than that of polydatin although the concentration of polydatin was higher than resveratrol in PCSE (**Figures 2B–D**). The results suggest polydatin may be transformed into resveratrol in the intestine of the mice. The results were consistent with an earlier report. Polydatin is considered as one of the main active ingredients of PCS and mainly exists in the root and stem of the PCS, whereas polydatin could be metabolized into resveratrol in intestine (Zhang et al., 2008). Thus, the effects of PCSE on a mouse UC model may be mainly caused by resveratrol and emodin.

**FIGURE 12:**
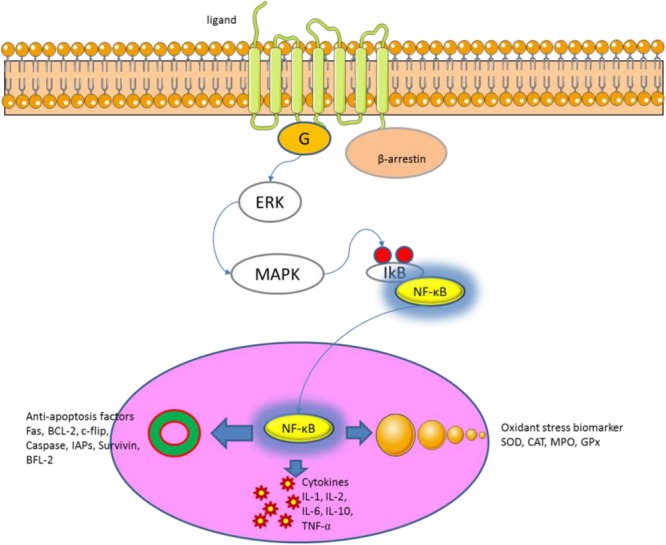
NF-Kb signaling pathway regulates cellular inflammatory, oxidative and apoptosis.

The etiology and pathogenesis of UC remain widely unclear. Most of them are considered to be related to microorganisms (Du et al., 2015; Coutinho et al., 2017), immune disorders (Baker and Isaacs, 2017; Rosen et al., 2017), environment (Wang et al., 2014; Petrakis et al., 2015) and genetics (Kopylov et al., 2016; Sifuentes-Dominguez and Patel, 2016). In recent years, the relationship between NF-κB and UC has become the focus of research (Eissa et al., 2017; Gu et al., 2017). NF-κB is a light chain of immunoglobulin kappa enhancing κB sequence-specific binding of nuclear protein transcription factors. Some studies have shown that continued inhibition of NF-κB will trigger immunodeficiency and peripheral lymphoid organ dysfunction (Stoffel et al., 2004; Scuto et al., 2013). NF-κB binds to IkB-α in the non-stimulated state and exists in an inactive state. IkB includes IkB-α, IkB-β and other family members. The main function of β-arrestin is to bind cell surface receptors and activate protein kinase, which phosphorylates IkB and dissociates it from the NF-κB complex, and releases NF-κB. Activated NF-κB complex translocates into the nucleus, combining with other transcriptional regulators to exert biological effects (**Figure 12**).

The NF-κB pathway has been proved to be the common pathway of inflammatory response and immunity and is an effective target for many diseases. Based on the above studies, the present experiment analyzed the expression of key molecules of NF-κB pathway in a mouse UC models. DSS treatment activates NF-κB signaling pathway, and results in the expression of inflammatory cytokines IL-1β, IL-6 and TNF-α, and down-regulation of IL-10. On the other hand, IL-1β also plays a regulatory role in the activation of NF-κB signaling pathway (Wang et al., 2015), and the up-regulation of inflammatory cytokines leads to pathological progress of UC.

PCSE is rich with phenolics and has antioxidant and anti-inflammatory activities (Kuo et al., 2013; Santomauro et al., 2017). PCSE can restore oxidant and reductant balance, repair intestinal bacteria barrier function, thereby inhibiting the growth of bacteria, reducing endotoxin content. PCSE exert inhibitory effects on the growth of *Streptococcus mutans* and *Streptococcus sobrinus* (Song et al., 2006). *P. cuspidatum* roots were found to be against common bacteria (*Bacillus cereus, Listeria monocytogenes, Staphylococcus aureus, Escherichia coli* and so on) (Shan et al., 2008). All these results suggest that they are beneficial to control bacterial diseases. PCSE has been proven to improve lipid composition of UC models by reducing MDA level and can improve cellular immune function by reducing the levels of IL-1β, IL-6, TNF-α and increase the level of IL-10 (**Figures 5, 7C**). TUNEL analysis showed that DSS treatment increased the apoptosis rates in a mouse UC model (**Figure 10**). In contrast, PCSE consumption reduced apoptosis rate with the increase in it dosage. On the other hand, PCSE inactivates NF-κB signaling pathway, which is found to be associated with the expression of BCL-2, whereas BCL-2 is an important anti-apoptosis protein (Xu et al., 2016; Yang et al., 2016). All the results suggest there may be other mechanisms for PCSE controlling apoptosis in UC mice.

The phenolic fraction has been found to ameliorate inflammatory responses by inactivating NF-κB and MAPKs (Chen et al., 2017). PCSE with rich phenolics may inhibit NF-κB pathway or block the entry of NF-κB into the nucleus, rendering it unable to activate cytokines, and thus PCSE acts as a potential anti-ulcerative colitis drug. Other studies have shown that PCSE can effectively reduce oxidative stress (Ghanim et al., 2010), which may be associated with UC risk. Present results demonstrate that PCSE can effectively reduce level MDA and activity of MPO and increase activity SOD, CAT and GPX (**Figure 8**), which will improve the antioxidant capabilities of UC mice.

To explore the exact molecular mechanism for the function of PCSE, the effects of each ingredients and combined ingredients on the cytokines and NF-κB related molecules were investigated by using the intestinal cells from a mouse UC model. The present findings demonstrated that the combination of polydatin, resveratrol or emodin, and PCSE exhibited higher inhibitory activities for these molecules than any one of the three components with same concentration treatment (**Figure 11**). The results suggest that the synergistic effects of polydatin, resveratrol or emodin on NF-κB signaling.

There are some limitations for the present work: (1) The ingredients of PCSE are complex and exact functional molecule remains unknown; (2) The present work only focuses on the association between different PCSE dosage and the changes of main molecules in NF-κB signaling pathway. Gene silence and overexpression should be performed to make the mechanism clearer; (3) considering the animal protection, the histology analysis was not performed in all groups before the present experiment. Therefore, to get more evidence for the present findings, further work is still needed in the future.

In sum, PCSE can effectively control UC development by improving antioxidant and anti-inflammatory capabilities of mouse UC models via NF-κB signaling pathway. Cell experiment demonstrates that PCSE may affect NF-κB signaling pathway via synergistic effects of polydatin, resveratrol or emodin. The present findings are promising and support the use of PCSE as a source of natural anti-inflammatory, anti-apoptosis and antioxidants. Regarding for the complex ingredients of PCSE and the present animal experiments, further work and clinical trials are highly demanded, and PCSE may be developed a potential drug for UC therapy in the future.

## Author Contributions

BL and XP: conceived and designed the experiments and wrote the paper. SL, XS, XL, HL, and TW: performed the experiments. LyG, LmG, SC, and YL: analyzed the data. XP: contributed reagents, materials, and analysis tools.

## Conflict of Interest Statement

The authors declare that the research was conducted in the absence of any commercial or financial relationships that could be construed as a potential conflict of interest.
